# Survey data on COVID-19-related fake news sharing on Facebook

**DOI:** 10.1016/j.dib.2026.112533

**Published:** 2026-01-31

**Authors:** Cristiane Melchior, Thierry Warin, Mírian Oliveira

**Affiliations:** a Department of Social Sciences, School of Engineering Sciences, Lappeenranta–Lahti University of Technology (LUT), Yliopistonkatu 34, Lappeenranta 53850, Finland; b Business School, Pontifical Catholic University of Rio Grande do Sul (PUCRS), 50th building, Av. Ipiranga, n° 6681, Porto Alegre, RS 90619-900, Brazil; c Department of International Business, École des hautes études commerciales de Montréal (HEC Montréal), 3000, chemin de la Côte-Sainte-Catherine, Montréal, Québec H3T 2A7, Canada

**Keywords:** Fake news, Covid-19, Survey, Facebook, Health crisis, Mixed method approach, Structural equation modeling (SEM), Fuzzy-set qualitative comparative analysis (fsQCA)

## Abstract

This data article describes the dataset derived from a survey investigating the factors associated with sharing fake news about COVID-19 on Facebook. The dataset comprises 338 valid responses collected between November 29, 2022, and January 17, 2023, primarily from users in Brazil. The questionnaire remained open until May 3, 2024, receiving 55 additional valid responses, which are provided as an additional resource. The data includes individual self-reported measures for Fake News Sharing (FNS), Trust (TR) in information on Facebook, Literacy Skills (LS), and Motivation of Friends to Share Information (MFSI) (including intrinsic and extrinsic constructs), alongside key demographic information such as age, gender, and education level. This resource is valuable for researchers in computational social science, crisis communication, and public health, as it enables quantitative and qualitative analysis of user behavior related to fake news, the role of self-assessed digital literacy, and the predictors of trust in unreliable sources during a global health crisis

Specifications Table

**Table:** 

Subject	Social Sciences
Specific subject area	Social media metrics, Data Science, computational methods, Post-COVID-19.
Type of data	Structured text data (Anonymizedand Filtered) in CSV and QS format.
Data collection	We collected a corpus of 468 answers from Facebook users using an online questionnaire administered via the Qualtrics XM software. The data collection period spanned 50 days, from November 29th, 2022, to January 17th, 2023. The questionnaire remained open until May 3, 2024, receiving 96 additional answers, which are provided as an additional resource. The target audience are comprised of Facebook users, aiming to investigate their motivations for sharing fake news about COVID-19. A snowball sampling technique was employed, where the survey link was shared across various Social Media Platforms (SMPs), including Facebook, and participants were encouraged to share it within their networks. The anonymous questionnaire was available in Portuguese, English, and French. Our study utilized previously validated scales, featuring 13 multiple-choice questions measured on a seven-point Likert scale, ranging from “strongly disagree” to “strongly agree.” To ensure data quality and validity, a rigorous treatment process was applied: from the 468 original answers, 97 incomplete answers were removed: 14 answers completed faster than the minimum reading threshold (2 min and 47 s) were excluded. We also removed five answers from respondents younger than 18. The final dataset comprises 338 valid responses. This dataset includes measurements for the constructs: Literacy Skills (LS), Trust (TR), Motivation for friends to Share Information (MFSI), and Fake News Sharing (FNS), alongside socio-demographic characteristics (age, gender, country of residency, and education level). The collected data enable advanced quantitative analyses using Structural Equation Modeling (SEM) and qualitative configurational analysis using fuzzy-set Qualitative Comparative Analysis (fsQCA) [1].
Data source location	Institution:Pontifical Catholic University of Rio Grande do Sul (PUCRS) City/Town/Region: Porto Alegre, Rio Grande do Sul Country: Brazil Latitude and Longitude (Data Storage and Processing Centers): 30°1′59.02″S 51°13′48″W Primary Data Sources: The dataset was collected using Qualtrics. The collected data were processed and stored in a secure research infrastructure maintained by PUCRS.
Data accessibility	Repository name: data_covid19_fake_news_facebook [2] Direct URL to data: https://github.com/crmelchior/data_covid19_fake_news_facebook Data identification number: DOI: 10.5281/zenodo.17904674
Related research article	The dataset presented in this article was collected and analyzed for the article: An investigation of the COVID-19-related fake news sharing on Facebook using a mixed methods approach [1], by Cristiane Melchior, Thierry Warin, and Mirian Oliveira, published in the journal Technological Forecasting & Social Change, vol 213, 2025, 123,969. DOI: https://doi.org/10.1016/j.techfore.2024.123969 For public availability, the original dataset has been anonymized and summarized. These adjustments safeguard participant confidentiality and enhance the dataset’s accessibility and usability for reuse by the broader research community. Comprehensive information regarding the original data collection is available in the previously cited publication.

## 1 Value of the Data

Our dataset offers a substantial contribution to research in digital sociology, health communication, and information systems, particularly within the context of social media behavior during a global health crisis, like the COVID-19. This dataset, comprising 338 unique filtered responses from a survey of Facebook users, focuses on self-reported behaviors and perceptions regarding the sharing of COVID-19-related fake news. It provides a unique and empirical resource for understanding the complex psychological and motivational factors driving fake news dissemination.

Our data is publicly available to ensure broad utility and reusability, provided that ethical guidelines regarding human subject data are respected. This release is supported by detailed documentation, which provides clear variable definitions, descriptive statistics, and methodological notes (including the application of post-stratification weights), promoting research transparency and facilitating replicability. For more details, see the full content of our article: An investigation of the COVID-19-related fake news sharing on Facebook using a mixed methods approach, DOI: https://doi.org/10.1016/j.techfore.2024.123969.

*The primary value of this data lies in the following areas:*

• (a) Provides a direct measure of self-reported susceptibility factors: Our dataset includes multi-constructs, validated scales for key behavioral and cognitive constructs: self-reported Literacy Skills (LS), Trust (TR) in information sources, Motivation for Friends to Share Information (MFSI) (categorized into intrinsic and extrinsic drivers), and Fake News Sharing (FNS) behavior. This allows researchers to test behavioral models (like the Theory of Planned Behavior or the Third-Person Effect) against real-world crisis data.
• (b) Enables configurational analysis using fsQCA: The data is optimized for use with qualitative comparative analysis (fsQCA), as demonstrated in the related research article. This enables scholars to identify complex, non-linear combinations of factors (configurations) that lead to the presence or absence of fake news sharing, offering a deeper, qualitative understanding beyond standard correlation-based modeling.
• (c) Focuses on a critical health communication crisis (COVID-19): The data captures user behavior and perceptions specifically during the intense period of the COVID-19 “infodemic,” offering insights into how user motivation and trust change when confronted with urgent, ambiguous, and life-altering health information. This is invaluable for future crisis communication studies.
• (d) Supports comparative methodological research: The related article demonstrated how the same dataset can be analyzed using multiple Structural Equation Modeling (SEM) approaches (CB-SEM vs. PLS-SEM) and software (R packages vs. SmartPLS). The anonymized data is therefore a valuable benchmark resource for methodological scholars testing new statistical modeling techniques or comparing the impact of data weighting on model fit.
• (e) Allows exploration of the Boomerang Effect: The inclusion of data on users’ beliefs regarding the “Boomerang Effect” (where corrective messages can backfire) allows researchers to study the interaction between a user’s self-assessed digital competence (LS) and their perception of how effective public health fact-checking interventions are on others.

## 2 Background

The COVID-19 pandemic triggered an "infodemic" on Social Media Platforms (SMPs), with Facebook identified as a primary vector for health-related fake news. This spread poses significant public health threats to public health and increasing anxiety [3,4], exacerbated by users often lacking the literacy skills required to verify content, effectively shifting the burden of factual accuracy from journalists to the general public [5,6].

This dataset addresses the critical need for empirical insight into the factors compelling Facebook users to share misinformation. The data collection was grounded in a dual-theoretical framework: Rumor Theory, which posits that rumors flourish amidst high importance and informational ambiguity to reduce anxiety [7] and the Boomerang Effect, where corrective interventions may inadvertently reinforce false beliefs [8].

Generated via a multi-language online survey, the dataset captures a conceptual model linking users’ Literacy Skills (LS), Trust (TR) in information sources, and the Motivation of Friends to Share Information (MFSI) to the likelihood of Fake News Sharing (FNS). This resource provides a vital foundation for behavioral research aimed at designing effective strategies to mitigate the spread of fake news during health crises.

## 3 Data Description

The dataset consists of 468 responses collected using an online survey, of which 338 were deemed valid for data analysis. The data collection took place from November 29th, 2022, to January 17th, 2023. The questionnaire remained open until May 3, 2024, and received 96 additional responses (55 of which are valid). The full dataset, along with the scripts used to filter and prepare the responses for analysis, is available in the repository. The anonymized dataset is a tabular file where each record (row) corresponds to an individual survey respondent. The columns contain responses to the questionnaire, which used a seven-point Likert scale (ranging from “strongly disagree” to “strongly agree”) for measuring the key constructs.

*The repository contains the following files:*

• data- anonymized-export-qualtrics.csv – Contains 564 raw anonymized answers exported from Qualtrics. Columns containing identifiable information, such as IP addresses, were removed, as were the exact start and end times of the response. The duration and date when the answer was recorded are available in the file.
• generate_data_preprocessed.R – Script in R language that takes the anonymized data exported from Qualtrics (see above) as input, filters valid answers (e.g., only finished answers), applies data preprocessing (e.g., standardize other country names), and exports the data preprocessed as CSV and QS (see below).
• data-preprocessed-cutoff-2023–01–17.csv (or .qs) – Contains 338 answers generated by the R script with cutoff date set as January 17th, 2023. This is the data originally used for the analysis in the journal article linked to this data paper.
• data-preprocessed-no-cutoff.csv (or .qs) – Contains 393 answers generated by the R script without a cutoff (55 more valid answers than the previous file).

Table 1 *presents the key attributes defined in each record of our dataset:*

**Table 1:** Attributes and their description.

Attribute	Description
UserLanguage	Language the respondent used in the questionnaire
Qgender	Gender of the respondent
Qage	Age range of the respondents
QCountry	Country of residency of the respondents
QCountry_4_TEXT	Other country that was not listed in the “QCountry” variable
Qeducation_level	Highest level of completed education of the respondent
Qarea	Areas of professional activity of the respondent
Qarea_7_TEXT	Other areas of professional activity of the respondent that were not listed in the “Qarea”
Qdigital_literacy	Self-reported ability to distinguish between false and true information
Qliteracy_scale_1 to 8	Rate of statements about respondent’ relation to the information about Covid-19 on Facebook on a scale from 1 to 7, where 1 means totally disagree and 7 means totally agree
Qagent	Who the respondent believe is the main responsible for correcting fake news about Covid-19 on Facebook
Qagent_5_TEXT	Other responsible for correcting fake news about Covid-19 on Facebook not listed in the “Qagent” variable.
Qboomerang	Respondents indicated whether they believe fake news corrections have the opposite effect
Qtrust_Qshare_1 to 5	Measures the individual’s self-reported tendency to trust and to share fake news on a scale of 1 to 7, where 1 means totally disagree and 7 means totally agree.
Qfrequency	How often the respondent share content about Covid-19 on Facebook
Qshare_you_1 to 8	Measures the individual’s self-reported tendency to share fake news on a scale of 1 to 7, with 1 being least relevant and 7 being most relevant.
Qshare_friends_1 to 8	Measures perceived intrinsic and extrinsic motivations for friends to share content on a scale of 1 to 7, with 1 being least relevant and 7 being most relevant
ResponseId	Sequential number to identify each answer
Qliteracy_scale_mean	Mean of the answers for the Qliteracy question
Qtrust_Qshare_mean	Mean of the answers for the Qtrust_Qshare_1 and Qtrust_Qshare_2 questions
fake_news_sharing_mean	Mean of the answers for the fake news sharing questions (Qtrust_Qshare_3 to 5)
Qshare_you_mean	Mean of the answers for the Qshare_you question
Qshare_friends_mean	Mean of the answers for the Qshare_friends question
Qshare_friends_im	Mean of the answers for the Qshare_friends intrinsic motivation statement
Qshare_friends_ex	Mean of the answers for the Qshare_friends extrinsic motivation statement

Source: theauthors, 2025.

The original questions presented to users in the questionnaire are available as supplemental material in the article published in the journal Technological Forecasting and Social Change: https://doi.org/10.1016/j.techfore.2024.123969.

## 4 Experimental Design, Materials and Methods

The dataset was collected using an online questionnaire designed to measure Facebook users’ self-reported behaviors and perceptions regarding COVID-19 fake news sharing. The instrument was based on pre-existing, validated scales from the literature, which were adapted to the context of COVID-19 fake news on Facebook. A rigorous process of reverse translation (English-Portuguese-English) and review by three external researchers was conducted to ensure grammatical correctness.

We focus on the analysis of the following constructs: (a) Literacy Skills (LS): adapted from Lee et al. [9] and Li et al. [10]; (b) Trust (TR), and Fake News Sharing (FNS): adapted from Chua and Banerjee [11], Khan and Idris [12], and Talwar et al. [13]. (c) Motivation of Friends to Share Information (MFSI): Classified into intrinsic and extrinsic motivations based on the self-determination theory taxonomy [14] and in our previous work [5].

The final publicly available dataset comprises 338 valid responses collected from Facebook users. The sample is geographically concentrated, with 80.77 % of respondents residing in Brazil, and is predominantly female (58.58 %). The respondents essentially represent a highly educated cohort, with the majority holding a college, specialization, or master’s degree, and falling within the 25 to 44 years old age bracket. The necessity to account for this specific demographic profile led to the use of post-stratification weights to ensure the reliability and validity of the structural equation modeling (SEM) analysis

### 4.1 Post-stratification weight

To mitigate potential bias introduced by the snowball sampling technique (since the sample was not perfectly representative of the target population, particularly with 80.77 % of respondents from Brazil), the data were calibrated using post-stratification weights. The weights were calculated in the R software using survey package. The calibration targeted three key variables: age, gender (from the general Facebook user population), and education level (from the Brazilian population). Weights larger than 4 were trimmed to maintain stability. This process ensures the resulting data, while self-reported, are statistically adjusted to approximate the characteristics of the target population for enhanced inferential validity.

## Limitations

While this dataset provides insights into the motivations associated with fake news sharing on Facebook, there are some limitations:

• Sampling bias: The data were collected through the researchers’ personal social networks, which may have introduced selection bias. Although post-stratification weighting was applied to mitigate this issue, the sample may not fully represent the broader population of social media users.
• Geographical concentration: A majority of respondents are from Brazil, which limits the dataset’s generalizability across different sociocultural and media contexts. Comparative studies using data from other countries are encouraged to validate and extend these findings.
• Platform specificity: The dataset focuses on Facebook. Since each social media has distinct user demographics and engagement patterns, future research should expand its scope to include other platforms.
• Self-reported measures: The variables related to literacy skills, trust, and fake news sharing behavior rely on self-reported data rather than observed behavior. Although prior literature supports the correlation between self-reported and actual sharing behavior, this reliance may still introduce perceptual bias.

Overall, while these constraints delimit the dataset’s scope, they do not diminish its analytical value. Instead, they highlight opportunities for future work to validate and extend the dataset across broader populations, multiple platforms, and behavioral tracking contexts.

## Ethics Statement

This study was conducted in strict accordance with institutional and international ethical guidelines for research involving human participants. Participation in the survey was voluntary and anonymous. Before beginning the questionnaire, all respondents were provided with a clear statement explaining the study’s objectives, the confidential treatment of their responses, and their right to withdraw at any point prior to submission. No personally identifiable information, such as names, email addresses, or social media handles, was collected at any stage.

In addition, to further protect participant privacy, all personal metadata from the Qualtrics raw data (including original response IDs, IP addresses, and geolocation details) was removed. These measures ensured strict adherence to data protection principles and complied with best practices recommended in the research ethics literature [15,16]. Consequently, the publicly released dataset excludes all sensitive or personally identifiable information. The collection, storage, and dissemination of data fully conform to the General Data Protection Regulation (GDPR) and other relevant data privacy frameworks.

Because the study relied exclusively on self-reported information voluntarily provided by adults through a standardized online questionnaire and did not involve personal or health-related data, it was deemed exempt from formal Institutional Review Board (IRB) review [17]. The authors affirm that no harm or risk was posed to participants.

## CRediT Author Statement

Cristiane Melchior: Conceptualization, Methodology, Investigation, Resources, Project administration, Writing – review & editing; Thierry Warin: Resources, Supervision, Project administration, Writing – review & editing; Mirian Oliveira: Writing – Supervision, Methodology, Validation, Data curation, Software, Writing – review & editing.

## Data Availability

10.5281/zenodo.17904674data_covid19_fake_news_facebook Public (Original data)
